# ^1^H-NMR-Based Endometabolome Profiles of *Burkholderia cenocepacia* Clonal Variants Retrieved from a Cystic Fibrosis Patient during Chronic Infection

**DOI:** 10.3389/fmicb.2016.02024

**Published:** 2016-12-20

**Authors:** Ana S. Moreira, Artur B. Lourenço, Isabel Sá-Correia

**Affiliations:** Department of Bioengineering, Institute for Bioengineering and Biosciences, Instituto Superior Técnico, Universidade de Lisboa (ULISBOA)Lisbon, Portugal

**Keywords:** *Burkholderia cepacia* complex, ^1^H-NMR based metabolomics, cystic fibrosis, chronic respiratory infection, stress tolerance, clonal variation, trehalose, glycine-betaine

## Abstract

During cystic fibrosis (CF) chronic lung infections, bacteria of the *Burkholderia cepacia* complex (Bcc) are exposed for several years to a stressful and changing environment. These environmental challenges results in genetic changes of the initial infecting strain with the consequent diversification of genotypes and phenotypes. The exploitation of functional and comparative genomic approaches has suggested that such diversification is associated with massive metabolic remodeling but these alterations are poorly understood. In the present work, we have explored a high resolution ^1^H-NMR-based metabolomic approach coupled to multivariate analysis to compare the endometabolome of three *B. cenocepacia* clonal variants retrieved from a CF patient from the onset of infection (IST439) until death with cepacia syndrome after 3.5 years (IST4113 and IST4134), to complement former proteomic and transcriptomic analyses. A fourth clonal variant (IST4129) retrieved from the same CF patient when the clinical condition worsened during the last months of life, was also examined since it was found to lack the third replicon. The metabolomic profiles obtained, based on the complete ^1^H-NMR spectra, highlight the separation of the four clonal variants examined, the most distinct profile corresponding to IST4129. Results indicate a variable content of several amino acids in the different isolates examined and suggest that glycolysis and the glyoxylate shunt are favored in late variants. Moreover, the concentration of two metabolites with demonstrated cellular protective functions against stress, glycine-betaine and trehalose, is different in the different isolates examined. However, no clear correlation could be established between their content and stress tolerance. For example, IST4113, previously found to be the most resistant variant to antimicrobials of different classes, exhibits low levels of trehalose and glycine-betaine but the highest resistance to heat and oxidative stress. Also, IST4129, with a high level of glycine-betaine but lacking the third replicon, previously associated with stress resistance and virulence, exhibits the highest susceptibility to all the stresses tested. Taken together, results from this study provide insights into the metabolic diversification of *B. cenocepacia* clonal variants during long-term infection of the CF airways.

## Introduction

Cystic fibrosis (CF) chronic lung infections caused by *Burkholderia cepacia* complex (Bcc) bacteria are usually associated with a rapid decline in lung function and decreased life expectancy (Mahenthiralingam et al., 2005; Lipuma, 2010). Once inside the CF lung, Bcc bacteria face a changing and stressful environment resulting from the activity of the host immune system, antimicrobial therapy, fluctuating levels of nutrients, decreasing oxygen availability and the presence of other co-infecting microbes (Döring et al., 2011). As proposed for *Pseudomonas aeruginosa* (Smith et al., 2006; Clark et al., 2015; Marvig et al., 2015), it is believed that these challenging environmental conditions stimulate genetic alterations in the initial infecting strain, leading to the diversification of genotypes and phenotypes and to the emergence of multiple clonal variants from the underlying Bcc population (Coutinho et al., 2011a,b; Lieberman et al., 2011, 2014; Moreira et al., 2014; Zlosnik et al., 2014; Miller et al., 2015; Maldonado et al., 2016).

The genome sequence and expression alterations experienced by Bcc bacteria during chronic infections have recently been on the focus of several studies. The genome sequences of late *Burkholderia cenocepacia* isolates retrieved from a CF patient after 10 years of chronic infection were compared with the initial infecting strain and mutations in genes involved in metabolism, including genes from amino acid, iron and purine metabolism, were identified (Miller et al., 2015). Another comparative genomic study focusing on *B. multivorans* isolates retrieved from a CF patient over 20 years of infection has shown that genes involved in gene expression regulation, cell envelope biogenesis, fatty acid and amino acid metabolism exhibit recurrent mutation patterns (Silva et al., 2016). Comparative genomic analysis of 112 *B. dolosa* isolates retrieved from 14 CF patients over 16 years of epidemic outbreak suggested that genes involved in secretion, outer membrane synthesis, oxygen-dependent regulation and antibiotic resistance are under strong selective pressure (Lieberman et al., 2011). Non-synonymous mutations in other genes involved in central metabolism were also detected, including five mutations in genes coding for three components of the 2-oxoglutarate dehydrogenase complex (Lieberman et al., 2011); two of these genes were also found to be mutated in a *B. cenocepacia* biofilm community after more than 1000 generations of selection (Traverse et al., 2013). The importance of cellular metabolism in Bcc persistence and pathogenesis was further supported by the demonstration of the crucial role of 21 metabolism-related genes in *B. cenocepacia* K56-2 survival (Hunt et al., 2004). Moreover, the comparison of genomic expression analyses based on quantitative proteomics and transcriptomics of three *B. cenocepacia* clonal variants retrieved from the same chronically infected CF patient during 3.5 years is also consistent with metabolism diversification during chronic CF lung infection (Coutinho et al., 2011a,b; Madeira et al., 2011, 2013; Mira et al., 2011). The three isolates examined in these genome-wide expression analyses include the first and the last isolates retrieved from the patient before death with the cepacia syndrome (IST439 and IST4134, respectively), and one intermediate isolate exhibiting a high resistance to several antimicrobial of different classes, retrieved after a period of exacerbated pulmonary function and intravenous therapy, IST4113. Several genes and proteins found to exhibit an altered expression in the two late isolates compared with the first isolate are involved in amino acid, purine, pyrimidine, tricarboxylic acid (TCA) cycle, glycolysis/gluconeogenesis, pyruvate metabolism, and oxidative phosphorylation. Taken together, these reports suggest a relevant role for metabolic reprogramming during Bcc persistence in the CF lung, even though the specific metabolic changes occurring along CF chronic lung infection are still poorly characterized.

Metabolomic approaches are of uttermost importance since metabolites are further down the line from gene to function, reflecting more closely the cellular activity at a functional level. However, studies involving this type of analysis are scarce, though the usefulness of a metabolomic approach for the understanding of bacterial metabolic adaptation was already demonstrated through the analysis of the osmotic stress effect in *B. cenocepacia* clinical isolates (Behrends et al., 2011) and the metabolomic footprinting of 179 *P. aeruginosa* isolates retrieved from several CF patients (Behrends et al., 2013). In the present work, we have performed an endometabolomic analysis of the three above referred *B. cenocepacia* clonal isolates (IST439, IST4113 and IST4134) whose genome-wide expression analysis was previously examined in our laboratory. This metabolomics analysis was based on ^1^H-NMR aiming to unveil the alterations occurring in the metabolome of these *B. cenocepacia* clonal variants during a chronic infection. This comparative analysis was extended to a fourth clonal variant (IST4129) retrieved from the same CF patient 3 months before death with the cepacia syndrome when the clinical condition worsened, given that it was found to lack the third replicon (Moreira et al., 2016). This replicon is required for virulence, tolerance to multiple stresses, D-xylose, pyrimidine and fatty acid utilization, as well as exopolysaccharide production and protease activity in some strains (Agnoli et al., 2012, 2014). Moreover, from the 776 predicted coding sequences of the *B. cenocepacia* J2315 third replicon, a high percentage corresponds to hypothetical proteins and genes with unknown function (Holden et al., 2009). Since our goal was to compare the data gathered in the present work for four previously characterized clonal variants (Cunha et al., 2003; Coutinho et al., 2011a; Moreira et al., 2016) retrieved from the same patient during chronic infection with the information previously obtained using other genome-wide expression approaches, cells were grown under conditions identical to those tested before and though to mimic those expected to occur during growth in the CF lung (Madeira et al., 2011, 2013; Mira et al., 2011). Although static measurement of metabolite pool sizes cannot be used to infer about metabolic fluxes, the observed changes may indicate potential alterations in pathway activity (Nandakumar et al., 2014). To the best of our knowledge, this is the first study using an endometabolomic approach coupled to multivariate statistical analysis to get insights into the metabolic diversity of *B. cenocepacia* during chronic infection of the CF airways.

## Materials and Methods

### Bacterial Isolates and Growth Conditions

The four *Burkholderia cenocepacia* clonal variants examined in this study, IST439, IST4113, IST4129 and IST4134, were recovered from the sputum of a CF patient under surveillance at the major Portuguese CF Center in the Hospital de Santa Maria from Centro Hospitalar Lisboa Norte EPE, Lisbon, from January 1999 to July 2002, as part of the hospital routine (Cunha et al., 2003; Coutinho et al., 2011a; Moreira et al., 2016). For each isolation date, one well isolated colony capable of growing onto the selective *B. cepacia* Selectatab medium was picked at random. Bacterial growth was carried out in Lysogeny Broth, Lennox (LB; Conda, Pronadisa), at 37°C and 250 rpm, or in LB agar plates obtained by supplementation of LB with 2% agar (Iberagar, Portugal).

### Endometabolome Profiling

#### Cell Sampling and Metabolite Extraction

*B. cenocepacia* isolates were grown in LB broth until mid-exponential phase (OD_640__nm_ of 0.4 ± 0.04). After dilution to a standardized OD_640__nm_ of 0.2 ± 0.02 in 0.9% NaCl (w/v), 100 μl of these cell suspensions were plated onto plates containing 30 ml of LB agar and then incubated for 24 h at 37°C. These growth conditions mimic those previously used to obtain cells to perform genome-wide expression analyses dedicated to three of the *B. cenocepacia* clonal isolates examined during the present study (Madeira et al., 2011, 2013; Mira et al., 2011), as well as the surface-attached growth expected to occur in the CF lung (Coutinho et al., 2011b; Madeira et al., 2011). Cells were washed from plates with 3 ml of 0.9% NaCl (w/v) and collected by centrifugation at 4500 × *g*, 4°C, during 10 min. The pellet was washed with 5 ml of 0.9% NaCl (w/v) and centrifuged again. One and a half ml of glass beads (0.4–0.6 mm diameter) and 9 ml of ethanol/water (75:25 v/v) heated to 80°C in a water bath were added to the pellets. The mixture was vortexed for 30 s, heated for 3 min to 80°C, vortexed again for 30 s and pelleted in a centrifuge cooled to -10°C (4500 × *g*, 10 min). The supernatant was transferred to a new tube and the extraction step was repeated with 6 ml of ethanol (75:25 v/v). The supernatants were mixed, cleared by centrifugation (10000 × *g*, -10°C, 10 min), dried under vacuum and stored at -80°C until NMR analysis. To increase signal sensitivity and reduce biological variation, two extracts from the same isolate and independently prepared were mixed together, producing a single sample (Malmendal et al., 2006; Colinet et al., 2013). For each isolate, six independent samples were analyzed by proton-NMR.

#### NMR Data Acquisition, Processing and Resonance Assignments

The dried extracts were dissolved in 1 ml of buffer (0.1 M phosphate buffer pH 7.00 in D_2_O, containing 0.1 M of d4-TSP) and transferred to 5 mm diameter NMR tubes. The samples were analyzed using a 500 MHz Avance III Bruker spectrometer, equipped with a 5 mm inverse probe (TXI), at 296 K, at the IST campus (Centro de Química Estrutural, CQE). One dimensional (1D) proton spectra were acquired using a train of CPMG echoes, of 40 ms long, to reduce macromolecules broad signal. All proton spectra were manually phased, baseline corrected and referenced to d4-TSP (δ 0.00 ppm) using Mnova v7.1 for NMR data processing. For metabolite identification purpose Total Correlation Spectroscopy (TOCSY; 512 × 2 K data points, relaxation delay of 2 s, mixing time of 60 ms and 64 transients per FID), and Heteronuclear Single Quantum Coherence (HSQC; 512 × 2 K data points, relaxation delay of 1.5 s, 64 transients per FID and a spectral width of 174 ppm in indirect dimension) spectra were collected for representative samples. Metabolite identity was assigned by careful analysis of chemical shifts, intensities, J couplings and multiplicities of the peaks present in 1D proton spectra, complemented with information from TOCSY and HSQC spectra and data gathered from different databases, as described previously (Lourenço et al., 2011, 2013). The identity of each metabolite was confirmed by comparison with spectra obtained for each authentic standard using the same sample conditions and NMR parameters.

#### Multivariate Data Analysis

The methodology used herein for the analysis of the NMR metabolic data has been extensively reviewed (Griffin, 2004; Trygg et al., 2007; Worley and Powers, 2013). Briefly, the spectra regions corresponding to residual water and ethanol were excluded (5.15-4.50, 3.73-3.63, 1.26-1.14 ppm). Each spectrum was integrated over a series of fixed width bins (0.005 ppm) using Mnova v7.1 features and normalized to its total intensity between 9.95 and 0.08 ppm. All spectra data were then put in a data table form in an ASCI format file and imported into the software SIMCA-P12^+^ for multivariate data analysis. The data was pre-processed in SIMCA-P12^+^ using Pareto scaling. PCA and O-PLS-DA were performed. Preliminary application of an unsupervised method like PCA provides an informative first look at the dataset structure and relationships between groups. After PCA, the supervised method O-PLS-DA was used to better discriminate between clusters evidenced by the PCA (Worley and Powers, 2016). SIMCA-P12^+^ provides a model summary table with different statistical measures for evaluating the model quality. Namely, the Sum of Squares of all the *x*-variables explained by the components (R^2^X), the Sum of Squares of all the y-variables explained by the components (R^2^Y) for the O-PLS-DA and the predicted power (Q^2^), calculated by sevenfold internal cross-validation, of the model. The *R*^2^ and *Q*^2^ should be similar. The value of Q^2^ ranges from 0 to 1 and typically a *Q*^2^ value higher than 0.4 is considered a good model, whereas if *Q*^2^ is substantially lower than *R*^2^ then the robustness of the model is poor (Westerhuis et al., 2008; Wheelock and Wheelock, 2013) For the O-PLS-DA modeling, a measure of the degree to which a particular variable explains cluster membership was obtained with the Variable Importance in the Projection (VIP) plot (with confidence intervals derived from jack knifing routine). Variables with VIP value above 1 are important for class membership.

#### Statistical Analysis

Statistical analysis was performed using Prism GraphPad software 6.05 (GraphPad Software, San Diego, CA, USA). To test if the data for each isolate and metabolite has a normal distribution, the Kolmogorov–Smirnov test was used. The statistical significance of the differences obtained in the relative abundance of each identified metabolite between the first isolate (IST439) and the late isolates was calculated using unpaired two tailed Student’s *t*-test for populations with Gaussian distribution, or the Mann–Whitney test for populations without Gaussian distribution. *P* ≤ 0.05 was considered statistically significant. Box and whisker plots were generated using GraphPad Prism 6.05 Software.

### Comparison of the Susceptibility of the Different *B. cenocepacia* Clonal Variants to Stress Conditions

#### Osmotic Stress

Liquid cultures grown in LB medium at 37°C and 250 rpm until mid-exponential phase were used to inoculate, at an initial OD_640_ of 0.05, 5 ml of LB broth supplemented with additional NaCl to a total concentration of 2 M or 3 M. Cultures were incubated in these media at 37°C and 250 rpm, and colony-forming units (CFUs) were determined by plating serial dilution of these cell suspensions in LB plates at different intervals. Results are the mean of at least three independent experiments.

#### Oxidative Stress

Liquid cultures grown in LB medium at 37°C and 250 rpm until mid-exponential phase were used to inoculate, at an initial OD_640_ of 0.05, 5 ml of LB broth supplemented with 0.01% H_2_O_2_. Cultures were incubated in this media at 37°C and 250 rpm, and CFUs were determined by plating serial dilutions of these cell suspensions in LB plates at different intervals. Results are the mean of at least three independent experiments.

#### Heat Stress

Liquid cultures grown in LB medium at 37°C and 250 rpm until mid-exponential phase were used to inoculate, at an initial OD_640_ of 0.05, 5 ml of LB broth. Cultures were incubated at 42°C or 44°C at 250 rpm, and CFUs were determined by plating serial dilutions of these cell suspensions in LB plates at different intervals. Results are the mean of at least three independent experiments.

### Ethics

Research studies involving the clinical Bcc isolates examined in this study and obtained as part of the HSM routine were approved by the hospital ethics committee and the patient’s anonymity is preserved.

## Results

### Alterations in the Endometabolome of *Burkholderia cenocepacia* Clonal Isolates Retrieved during Chronic Infection

**FIGURE 1:**
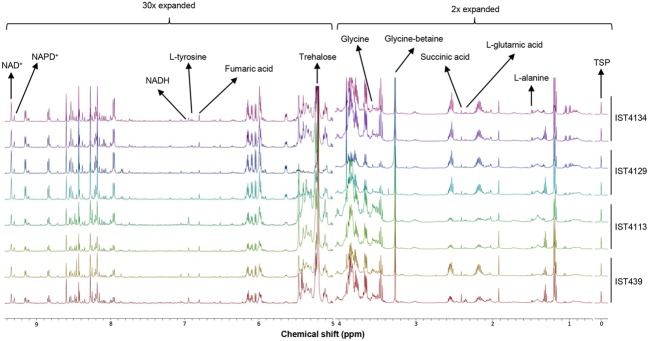
**Representative endo-metabolome profiles.** High resolution ^1^H NMR spectra from the endometabolomes of *B. cenocepacia* sequential clonal variants retrieved from a CF patient chronically colonized for 3.5 years. Spectra are representative of all the replicates obtained for each isolate and were normalized to the reference, TSP (δ = 0.0 ppm).

The ^1^H-NMR spectra acquired from extracts obtained from *B. cenocepacia* cells grown 24 h at 37°C onto the surface of LB agar medium allowed the detection of hundreds of resonances (**Figure 1**). Several classes of biomolecules were identified, including amino acids (L-alanine, L-valine, L-methionine, L-serine, glycine, L-glutamic acid, L-glutamine, L-aspartic acid, L-tyrosine, L-phenylalanine), osmolytes (trehalose and glycine-betaine), cofactors (NADH and NAD^+^), organic acids (acetic, fumaric and succinic acids), and metabolites associated with energy metabolism (ADP and AMP) (**Figure 1**, Supplementary Table S1).

**FIGURE 2:**
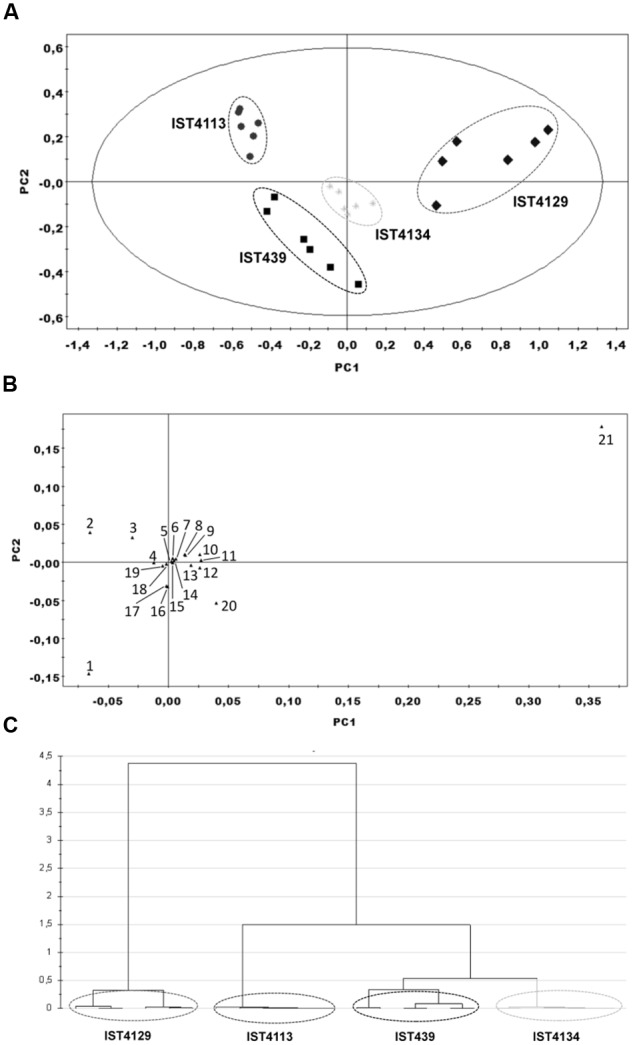
**Endometabolome multivariate analysis of four *B. cenocepacia* sequential clonal variants retrieved from a CF patient chronically infected for 3.5 years. (A)** 2D score plot displaying the space formed by the two first principal components (R^2^X for PC1 equal to 0.693 and R^2^X for PC2 equal to 0.139), **(B)** 2D loading plot displaying the space formed by the two first principal components and displaying the characteristic bins for a set of metabolites, and **(C)** dendrogram based on the PCA scores (ward clustering distance measure) considering all principal components of the model. For the 2D score plot shown in **(A)**, each point represents a spectra obtained for independent samples, resulting in six samples for each isolate. *Key*: (1) Trehalose, (2) Glycine, (3) L-serine, (4) Glucose, (5) Uracil, (6) Fumarate, (7) L-aspartic acid, (8) Oxidized glutathione, (9) AMP, (10) L-alanine, (11) NAD^+^, (12) L-valine, (13) ADP, (14) L-tyrosine, (15) NADP^+^, (16) Succinic acid, (17) L-glutamic acid, (18) L-phenylalanine, (19) NADH, (20) L-methionine, and (21) Glycine-betaine.

Modulation of the endometabolome data using PCA generated a model with four principal components (PCs) (R^2^X = 0.911 and *Q*^2^ = 0.862) (**Figure 2A**). The different biological replicates were grouped in four distinct clusters, which correspond to the four isolates under analysis, IST439, IST4113, IST4129 and IST4134 (**Figures 2A,C**). The analysis of both the score plot and dendrogram shows that the most distinct metabolic profile belongs to IST4129. Further analysis of this data using Orthogonal-Partial Least Squares-Discriminant Analysis (O-PLS-DA), and in accordance with the PCA modeling, clearly distinguished the different isolates under study (Supplementary Table S2), indicating that each of the four isolates is metabolically distinct from the others. Additionally, the loading plot reveals that glycine-betaine, trehalose, L-methionine, L-glycine, and L-serine were the metabolites that contributed more to cluster separation (**Figure 2B**). VIP analysis showed that glycine-betaine, trehalose, and glycine are three of the most important identified metabolites contributing to separate IST439 from IST4113 and IST4129, while glycine-betaine, L-valine, and succinic acid are the three identified metabolites that contribute more to separation between IST439 and IST4134. This analysis also revealed a significant contribution of a still unidentified metabolite, at 2.18 ppm, for separation between IST439 and IST4113.

**FIGURE 3:**
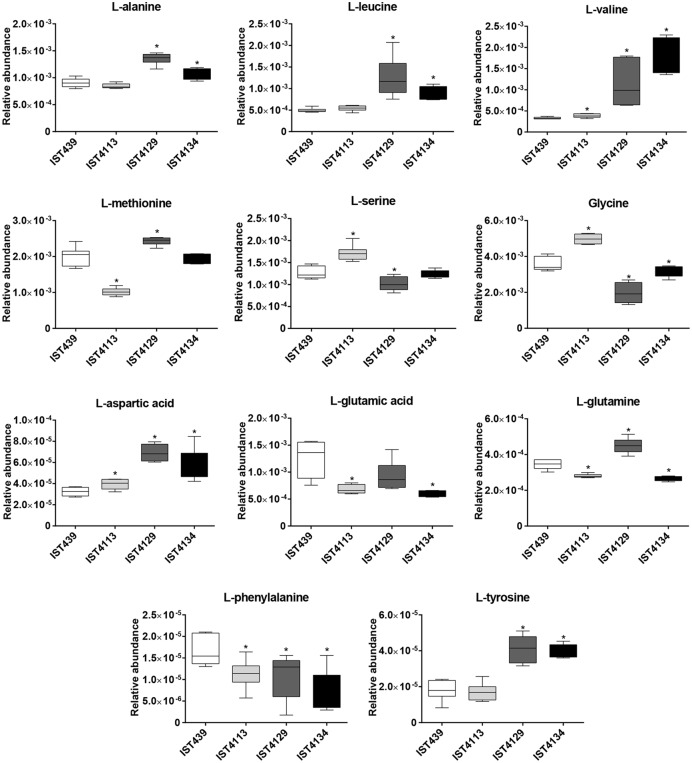
**Relative abundance of the amino acids detected through the comparative analysis of the endometabolomes of four *B. cenocepacia* sequential clonal variants retrieved from a CF patient chronically infected for 3.5 years.** Each plot shows the variation of the relative abundance of a representative bin in all the spectra for each metabolite and *B. cenocepacia* isolate. Median values are represented by the line in the middle of the box with the ends showing the 25th and 75th percentiles. Upper and lower ends of the lines represent the maximum and minimum values respectively. ^∗^*P* < 0.05 when each late *B. cenocepacia* isolate was compared to the first isolate, IST439.

**FIGURE 4:**
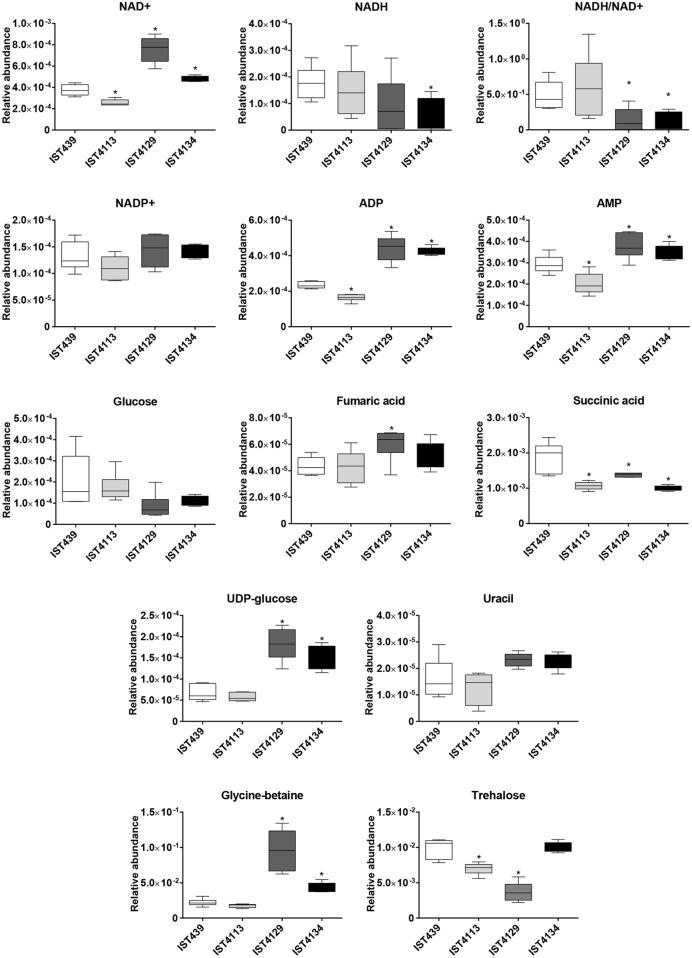
**Relative abundance of cofactors, metabolites involved in energy metabolism, organic acids, sugars, uracil and osmolytes, detected through comparative analysis of the endometabolomes of four *B. cenocepacia* sequential clonal variants retrieved from a CF patient chronically infected for 3.5 years.** Each plot shows the variation of the relative abundance of a representative bin in all the spectra for each metabolite and *B. cenocepacia* isolate. Median values are represented by the line in the middle of the box with the ends showing the 25th and 75th percentiles. Upper and lower ends of the lines represent the maximum and minimum values respectively. ^∗^*P* < 0.05 when each late *B. cenocepacia* isolate was compared to the first isolate, IST439.

Overall, no isolation time-dependent pattern of alteration in the relative abundance of several metabolites was identified (**Figures 3** and **4**). However, late isolates IST4129 and IST4134 showed higher levels of L-alanine, L-valine, L-tyrosine, L-aspartic acid, glycine-betaine, NAD^+^, ADP and AMP than the isolates retrieved before from the patient. Moreover, the levels of succinic acid and L-glutamic acid were higher in the first isolate retrieved from the patient, compared with the three late isolates. A metabolite-to-metabolite correlation analysis of the endometabolome data from each late isolate and isolate IST439 suggested that some metabolite concentration changes may be linked, namely succinic acid with L-glutamate, NAD^+^ with glycine-betaine, L-tyrosine and L-leucine, though with different *R*^2^ values (Supplementary Figure S1).

### Comparison of *B. cenocepacia* Clonal Variants Tolerance to Multiple Stresses

**FIGURE 5:**
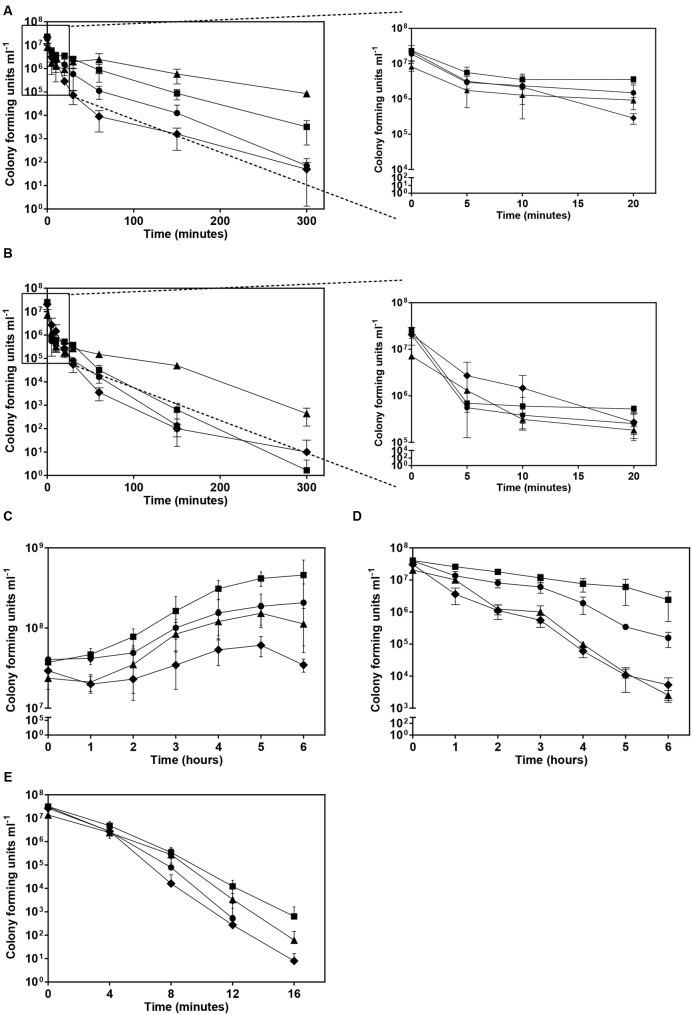
**Effect of different stresses in the viability of the cultures of four *B. cenocepacia* sequential clonal variants examined.** Cultures of the four isolates under study, IST439 (▲), IST4113 (■), IST4129 (◆) and IST4134 (●) cultivated in the absence of stress were exposed to different stresses, namely osmotic (saline) stress using 2M NaCl **(A)** or 3M NaCl **(B)**, heat stress at 42°C **(C)** or 44°C **(D)**, and oxidative stress using 0.01% hydrogen peroxide **(E)**. Error bars represent standard deviation.

Since glycine-betaine and trehalose levels were found to exhibit significant differences between the *B. cenocepacia* clonal variants examined (**Figure 4**) we have compared the ability that the different variants grown in the absence of stress have to survive to sudden exposure to heat, oxidative or osmotic (saline) stresses (**Figure 5**). In fact, these solute compatible compounds have been implicated in *B. cenocepacia* response to osmotic (saline) stress (Behrends et al., 2011) and shown to have additional roles in microbial protection against temperature and oxidative stress (Caldas et al., 1999; Canovas et al., 2001; Alvarez-Peral et al., 2002; Liu et al., 2011; Pilonieta et al., 2012).

In general, isolate IST4129, lacking the third replicon previously described as being involved in resistance to multiple stresses (Agnoli et al., 2014) but exhibiting the highest abundance of glycine-betaine and the lowest abundance of trehalose, was found to be the most susceptible clonal isolate to all the stresses tested (**Figure 5**). However, a slightly higher tolerance was apparently observed during the first 10 min following sudden exposure to osmotic stress with 3M NaCl (**Figure 5B**). Remarkably, whereas IST439 was the isolate with the highest tolerance to osmotic stress (**Figures 5A,B**), IST4113, which was the isolate with lowest levels of both glycine-betaine and trehalose, exhibits the highest tolerance to heat and oxidative stresses (**Figures 5C–E**).

## Discussion

The multivariate analysis of the metabolic profiles of the four *Burkholderia cenocepacia* clonal variants examined in this work shows that isolate IST439, the first isolate retrieved from the patient, is metabolically closer to the last clonal variant retrieved from the patient, IST4134, than to clonal variant IST4113 retrieved 9 months before the patient’s death and previously found to be remarkably resistant to antimicrobials of different classes (Coutinho et al., 2011a). Consistent with this conclusion is the expression proteomic data previously obtained for isolates IST4113 and IST4134 that also indicates that the number of proteins exhibiting different content in IST439 compared with the two late isolates is lower for IST4134 than for IST4113, with several proteins showing an altered content in IST4113, but not in IST4134, compared to IST439 (Madeira et al., 2013). The endometabolome profile analysis also showed that the clonal variant IST4129 lacking the third replicon (Moreira et al., 2016), exhibits the most distinct metabolic profile. Even though IST4129 and IST4134 were retrieved in the last stages of the illness at isolation dates separated by only 9 months (Coutinho et al., 2011a), it is intriguing why the loss of such a large genomic element in IST4129 does not significantly change the relative abundances of several of the metabolites that were identified in these isolates. However, this appears to be consistent with the conclusions from a previous transcriptomic analysis comparing *B. cenocepacia* H111 with the derived mutant lacking the third replicon that has shown that only one half of the third replicon genes are expressed in LB medium and only a few genes from chromosomes 1 and 2 are regulated by this megaplasmid (Agnoli et al., 2012). Moreover, the megaplasmid is essentially required for secondary metabolism, virulence and stress resistance of Bcc bacteria although a large percentage of the encoded genes are of unknown function (Holden et al., 2009; Agnoli et al., 2012, 2014). Our data also reinforce the idea that core functions, such as energy and central metabolism are not significantly affected by the loss of the third replicon and suggest that the separation between IST4129 and the other isolates based on multivariate analysis results from differences in parts of the spectra related with still unidentified metabolites (e.g., singlet at 3.905 or a doublet of doublets at 7.96).

**FIGURE 6:**
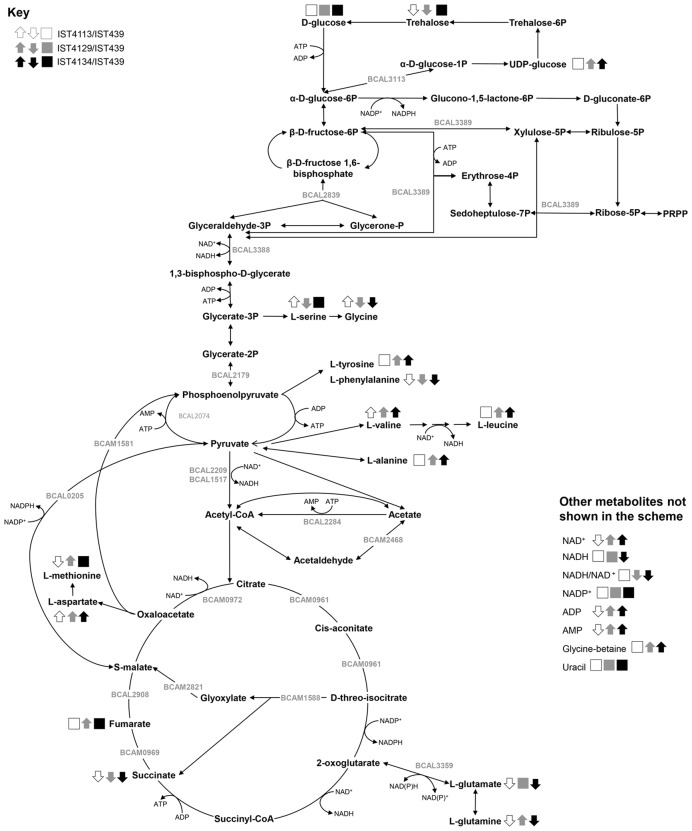
**Integrative and schematic overview of the metabolite changes detected in *B. cenocepacia* clonal isolates retrieved from the same CF patient.** Statistically significant changes in the identified metabolites that were observed (white, gray, and black arrows) or not (white, gray, and black boxes) between the first isolate retrieved from the patient, IST439, and the late isolates IST4113 (white symbols), IST4129 (gray symbols) and IST4134 (black symbols) are shown (see key at the top left of the figure). The locus name of some of the genes and proteins with altered content in at least one of the late isolates compared with IST439 are also shown. This metabolic scheme was based on information gathered from KEGG PATHWAY Database (http://www.genome.jp/kegg/pathway.html), in BioCyc Database Collection (http://biocyc.org/) and in the metabolic reconstruction of *B. cenocepacia* J2315 (Bartell et al., 2014).

Results from the identified changes occurring in the endometabolome of *B. cenocepacia* clonal variants retrieved from a CF patient chronically infected during 3.5-years, from the onset of the infection until death with the cepacia syndrome, are summarized in **Figure 6**. Marked differences in the level of the pyridine nucleotides NADH and NAD^+^ were observed between the first isolate retrieved from the patient and the late isolates IST4129 and IST4134. Since these cofactors are the most important redox carriers involved in metabolism, acting as electron acceptors in catabolic processes and providing the necessary reducing power for energy-producing redox reactions, having been proposed as involved in more than 120 reactions throughout *B. cenocepacia* metabolism (Fang et al., 2011; Bartell et al., 2014), it is tempting to speculate that the altered redox status of these isolates might contribute to several of the metabolite differences observed. Among the alternative pathways that can be used in the turnover of NAD^+^ to NADH is the biosynthesis of glycine-betaine, L-tyrosine and L-leucine from betaine aldehyde, chorismate and valine, respectively (Bartell et al., 2014). Remarkably, the relative abundance of glycine-betaine, L-tyrosine and L-leucine is higher in IST4129 and IST4134 compared with IST439. Differently, isolate IST4113 has a lower NAD^+^ and NADH content compared with IST439 but for NADH the difference is not statistically significant. In fact, the NADH/NAD^+^ ratio in IST4113 is not clearly distinct from the same ratio in IST439, as observed for several other changes found between the first isolate and the late isolates IST4129 and IST4134 but not for IST4113.

A reduction in the NADH/NAD^+^ ratio was registered in isolates IST4129 and IST4134 compared to the first isolate IST439, particularly due to the higher abundance of NAD^+^. In *Escherichia coli*, a low NADH/NAD^+^ ratio was shown to favor the metabolic flux toward the pyruvate dehydrogenase complex (PDHc) (de Graef et al., 1999), consistent with the upregulation of the expression of PDHc and several proteins involved in TCA registered in the late isolate IST4134 compared to IST439, as indicated by a previous expression proteomic analysis (Madeira et al., 2013). Also, the higher levels of fumaric acid and of the amino acid L-aspartate, derived from the intermediate of TCA cycle oxaloacetate, in both IST4129 and IST4134, suggest that the metabolism through glycolysis and TCA might be favored in these late isolates compared with the first isolate. The adaptation of *Pseudomonas aeruginosa* to the CF lung environment was previously proposed to occur through significant metabolic changes that include increased expression of genes involved in the TCA cycle and a reduction in acetate excretion, presumably resulting from changes that favor a more efficient utilization of carbon substrates and, consequently, a higher metabolic fitness in the CF lung environment (Hoboth et al., 2009; Behrends et al., 2013). The changes found in our study in late *B. cenocepacia* clonal variants IST4129 and IST4134 may presumably reflect a similar adaptive response to the CF lung environment.

An intriguing result was obtained for succinic acid, whose abundance is lower in all the late isolates compared to IST439, with no correlation with the level of the other identified intermediate of the TCA cycle or with L-aspartic acid. However, a similar trend could be observed for L-glutamic acid, an amino acid that can be synthesized from 2-oxoglutarate in a reversible reaction using the enzyme glutamate dehydrogenase (BCAL3359), whose expression is down-regulated in IST4113 compared to IST439 (Mira et al., 2011). Also, in the late isolates IST4113 and IST4134 retrieved from the patient, there is an increase in the expression of the enzymes involved in the glyoxylate shunt (Madeira et al., 2011, 2013; Mira et al., 2011), an anaplerotic pathway of the TCA cycle that allows bacterial growth using acetate or fatty acids as carbon source (Berg et al., 2002). These results suggest that the metabolic flux is diverted from the decarboxylating steps of the TCA to the glyoxylate shunt in late *B. cenocepacia* isolates. Remarkably, 2-oxoglutarate dehydrogenase, an enzyme that catalyzes the conversion of 2-oxoglutarate into succinyl-CoA in the TCA cycle, was previously found to be under strong selection pressure, either during adaptation of a single biofilm community of *B. cenocepacia* by more than one thousand generations of selection (Traverse et al., 2013) or in a retrospective study of a *B. dolosa* outbreak among CF patients that included 112 isolates collected over 16 years (Lieberman et al., 2011). The upregulation of the glyoxylate shunt was already reported to occur in late *P. aeruginosa* isolates retrieved from CF patients (Hoboth et al., 2009), and suggests the prevalence of metabolism of acetyl-CoA produced by β-oxidation from lipids in late Bcc and *P. aeruginosa* isolates. Remarkably, it was reported that blocking the TCA cycle in its decarboxylation steps results in increased *E. coli* survival to ampicillin exposure due to the reduction of the levels of reactive oxygen species (ROS) formed through a rapid NADH consumption after antibiotic treatment (Kohanski et al., 2007). The involvement of isocitrate lyase, the first enzyme of the glyoxylate cycle, in the *Mycobacterium tuberculosis* resistance to three clinically used tuberculosis drugs (isoniazid, rifampicin and streptomycin) was also demonstrated (Nandakumar et al., 2014) and the inhibition of this enzyme was shown to reduce the survival of *B. cenocepacia* persister cells in biofilms after treatment with tobramycin, a ROS-producing antibiotic (Van Acker et al., 2013). These results suggest that the upregulation of the glyoxylate shunt might limit the production of NADH through the bypass of the decarboxylating steps of the TCA and, consequently, the production of ROS in the late *B. cenocepacia* isolates under study. This can act as a protective mechanism against the antimicrobial therapy administered to the patient. Consistent with this hypothesis is the fact that the late *B. cenocepacia* isolates IST4113, IST4129 and IST4134 are more resistant to tobramycin than IST439 (Coutinho et al., 2011a) and that tobramycin was found to induce bacterial cell death through the production of ROS (Van Acker et al., 2013).

The high availability of amino acids in the CF sputum was proposed to represent a potential driving force for bacterial adaptation to the lung environment (Thomas et al., 2000; Schwab et al., 2014). Consistent with this proposal is the fact that several studies report the emergence of CF isolates of Bcc and *P. aeruginosa* that are auxotrophic for specific amino acids (Barth and Pitt, 1995a,b; Thomas et al., 2000) and distinct lineages of late *P. aeruginosa* isolates were shown to use more efficiently amino acids with higher biosynthetic cost than early isolates (Behrends et al., 2013). Results from the present work reveal changes in the pool of several amino acids between the sequential *B. cenocepacia* isolates, but no association with their metabolic cost could be established since the differences observed likely result from a balance between catabolism and anabolism of each amino acid rather than essentially from differences in the ability of each isolate to uptake amino acids from the medium and metabolize them. Also, late isolates are not amino acid auxotrophs since their growth curves are similar when grown in the basal-mineral M9 medium, lacking amino acids (unpublished data).

Although the metabolomic analysis here described was not performed under stress conditions, metabolites with a described protective function against stress were identified as having different abundances in the *B. cenocepacia* clonal variants under study. Two of these metabolites, glycine-betaine and trehalose, were implicated in *B. cenocepacia* protection against osmotic stress (Behrends et al., 2011). Additional roles of these compatible solutes in protection against temperature and oxidative stress were also described in other microorganisms (Caldas et al., 1999; Canovas et al., 2001; Alvarez-Peral et al., 2002; Liu et al., 2011; Pilonieta et al., 2012). However, results from this study did not indicate a clear correlation between the abundance of glycine-betaine and/or trehalose with *B. cenocepacia* tolerance against various stresses, including osmotic stress, even during early exposure to stress of cells grown in its absence. For example, given the low abundance of both trehalose and glycine-betaine in isolate IST4113, it was expected that it could exhibit a higher susceptibility to several stresses. However, this was the isolate with the highest resistance to oxidative and heat-induced stresses. Increased resistance to oxidative stress might be an important for Bcc survival during CF lung infection since this environment is characterized by increased ROS production by polymorphonuclear leukocytes (PMNs) and impaired antioxidant protection (Roum et al., 1993; Hull et al., 1997). Remarkably, IST4113 was the clonal variant retrieved from this patient with the highest resistance to all the antimicrobials previously tested (Coutinho et al., 2011a), some of which are known to induce oxidative stress (Kohanski et al., 2007; Van Acker et al., 2013). It is thus likely that this isolate may exhibit a higher resistance to oxidative stress due to mechanisms of ROS detoxification that do not involve trehalose or glycine-betaine. Additionally, the upregulation of the glyoxylate shunt in this isolate, already suggested by quantitative proteomic analysis (Madeira et al., 2011) and further supported by the metabolomic data obtained in this work, may contribute to increased resistance of this isolate to oxidative stress and antimicrobials due to decreased ROS generation. Other mechanisms unraveled by proteomic and transcriptomic analyses were already implied in the higher antimicrobial resistance of IST4113 compared to IST439, including a higher expression of proteins/genes involved in protein synthesis, translation, protein folding as well as multidrug efflux pumps, and a lower expression of porins in this late isolate (Madeira et al., 2011; Mira et al., 2011). Another remarkable result concerns isolate IST4129, which exhibits the highest level of glycine-betaine but also the highest susceptibility to all the tested stresses. However, since this isolate lacks the third replicon, a genomic element previously shown to be essential for stress resistance (Agnoli et al., 2014), its higher susceptibility to stress is not unlikely. The *in vivo* loss of this third replicon is a rare event (Agnoli et al., 2014; Moreira et al., 2016), and the loss of this megaplasmid involved in stress resistance, virulence and secondary metabolism is not expected to confer any advantage in the highly stressful CF environment. Thus, it is tempting to speculate that the higher abundance of glycine-betaine in isolate IST4129 could be a competitive factor contributing to the persistence of this clonal variant in the CF airways and, consequently, to its isolation.

## Conclusion

These results provide interesting insights into the metabolic versatility and diversity of *B. cenocepacia* clonal variants and reinforce the concept that *B. cenocepacia* diversify and adjusts metabolism when exposed to this challenging host environment during chronic lung infection in CF patients. However, these results should be interpreted with caution. Several recent papers are supporting the idea of the occurrence of a high Bcc phenotypic and genotypic diversity within a CF patient, where multiple lineages coexist presumably as the result of bacterial exposure to the dynamic CF lung environment during chronic infection (Coutinho et al., 2011a; Lieberman et al., 2014; Moreira et al., 2014, 2016; Miller et al., 2015). However, the *B. cenocepacia* clonal isolates examined in this work may not be representative of the expected population heterogeneity actually present at each isolation time in the CF airways. Only the extension of this metabolomic analysis to other Bcc clonal variants, strains and CF patients may provide more definitive conclusions concerning metabolic diversification and adaptive adjustment of *B. cenocepacia* to the CF lung during long term infections.

## Author Contributions

AM prepared the cell cultures, performed the metabolite extractions and the stress tolerance assays. AM and AL performed the ^1^H-NMR spectra acquisition and processing, and the multivariate analysis. AM prepared the figures and contributed to the writing of the manuscript under the scientific supervision of IS-C, who conceived and coordinated the study. All authors read and approved the final manuscript.

## Conflict of Interest Statement

The authors declare that the research was conducted in the absence of any commercial or financial relationships that could be construed as a potential conflict of interest.

## Abbreviations

Bcc: *Burkholderia cepacia* complex
CF: cystic fibrosis
CPMG: Carr-Purcell-Meiboom-Gill
d4-TSP: trimethylsilyl-2,2,3,3-tetradeuteropropionate
^1^H-NMR: proton nuclear magnetic resonance
HSM: Hospital de Santa Maria
O-PLS-DA: orthogonal partial least squares discriminant analysis
PCA: principal component analysis
ROS: reactive oxygen species
TCA: tricarboxylic acid cycle
VIP: variable importance projection
