# Supplementary material for: 1H-NMR-Based Endometabolome Profiles of Burkholderia cenocepacia Clonal Variants Retrieved from a Cystic Fibrosis Patient during Chronic Infection
Source: Front Microbiol. 2016 Dec 20;7:2024. doi: 10.3389/fmicb.2016.02024 (PMC5167703; doi:10.3389/fmicb.2016.02024)
Supplement: Supplementary file 1 [file Table_1.PDF]

Table S1. Metabolites identified in *Burkholderia cenocepacia* isolates.

Metabolite	Chemical shift (ppm) and multiplicity of a characteristic peak	Spectral data	Comparison with supplemented spectra
**L-leucine**	0.856 (doublet)	1D, HSQC and TOCSY	Yes
**L-valine**	0.996 (doublet)	1D, HSQC and TOCSY	Yes
**L-alanine**	1.485 (doublet)	1D, HSQC and TOCSY	Yes
**Acetic acid ***	1.921 (singlet)	1D and HSQC	Yes
**L-methionine**	2.142 (singlet)	1D, HSQC and TOCSY	Yes
**L-glutamic acid**	2.358 (multiplet)	1D, HSQC and TOCSY	Yes
**Succinic acid**	2.408 (singlet)	1D	Yes
**L-glutamine**	2.458 (multiplet)	1D, HSQC and TOCSY	Yes
**L-aspartic acid**	2.801 (doublet of doublets)	1D, HSQC and TOCSY	Yes
**Glycine-betaine**	3.271 (singlet)	1D, HSQC and TOCSY	Yes
**Glycine**	3.564 (singlet)	1D	Yes
**L-serine**	3.973 (multiplet)	1D and TOCSY	Yes
**Glucose**	4.662 (doublet)	1D	Yes
**Trehalose**	5.20 (doublet)	1D, HSQC and TOCSY	Yes
**UDP-glucose**	5.610 (doublets of doublets)	1D	Yes
**Fumaric acid**	6.522 (singlet)	1D	Yes
**L-tyrosine**	6.905 (multiplet)	1D and TOCSY	Yes
**NADH**	6.950 (doublet)	1D	Yes
**L-phenylalanine**	7.335 (doublet)	1D	Yes
**Uracil**	7.545 (doublet)	1D	Yes
**Formic acid ***	8.460 (singlet)	1D	Yes
**ADP**	8.539 (singlet)	1D	Yes
**AMP**	8.601 (singlet)	1D	Yes
**NADP+**	9.301 (singlet)	1D	Yes
**NAD+**	9.341 (singlet)	1D	Yes

* Volatile
