# Supplementary material for: 1H-NMR-Based Endometabolome Profiles of Burkholderia cenocepacia Clonal Variants Retrieved from a Cystic Fibrosis Patient during Chronic Infection
Source: Front Microbiol. 2016 Dec 20;7:2024. doi: 10.3389/fmicb.2016.02024 (PMC5167703; doi:10.3389/fmicb.2016.02024)
Supplement: Supplementary file 2 [file Table_2.PDF]

Table S2. O-PLS-DA modelling of the endometabolome data from patient J isolates. Summary of the statistics of the O-PLS-DA models of the metabolic profiles obtained for the sequential *B. cenocepacia* isolates retrieved from the patient J. Number of components (Principal components + Orthogonal components + Uncorrelated components).

		**Model statistics**		
**Comparison**	**Number of components**	**R^2X**	**R^2Y**	**Q^2**
**IST439, IST4113, IST4129 and IST4134**	3+1+0	0.908	0.928	0.88
**IST439 vs IST4113**	1+2+0	0.846	0.995	0.962
**IST439 vs IST4129**	1+0+0	0.779	0.877	0.856
**IST439 vs IST4134**	1+2+0	0.826	0.992	0.968
**IST4113 vs IST4129**	1+2+0	0.922	0.998	0.977
**IST4113 vs IST4134**	1+0+0	0.806	0.963	0.952
**IST4129 vs IST4134**	1+1+0	0.848	0.976	0.895
