# Supplementary material for: 1H-NMR-Based Endometabolome Profiles of Burkholderia cenocepacia Clonal Variants Retrieved from a Cystic Fibrosis Patient during Chronic Infection
Source: Front Microbiol. 2016 Dec 20;7:2024. doi: 10.3389/fmicb.2016.02024 (PMC5167703; doi:10.3389/fmicb.2016.02024)
Supplement: Supplementary file 3 [file Image_1.PDF]

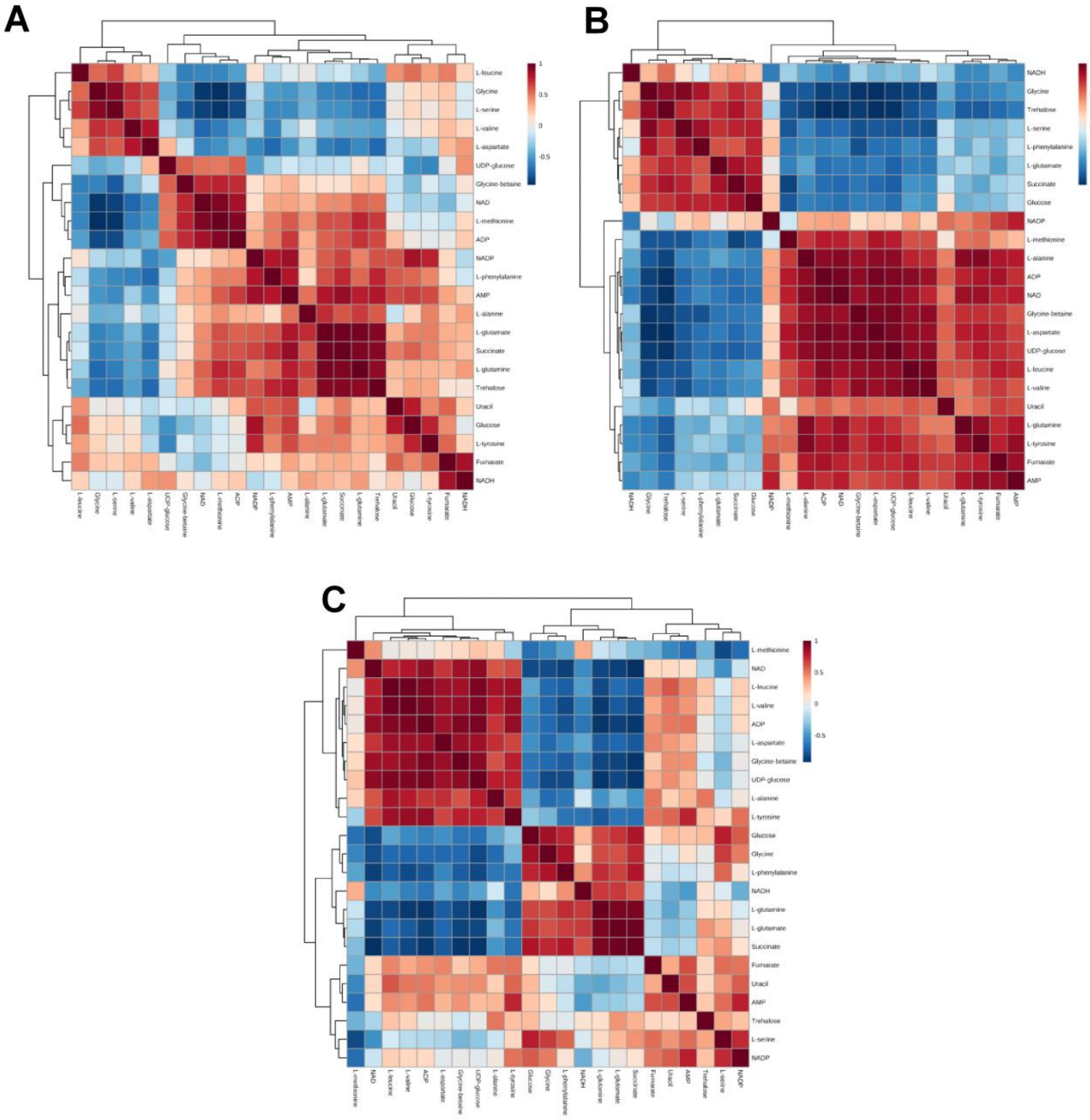

Figure S1. Metabolite to metabolite correlation analysis based on the endometabolome changes detected in *B. cenocepacia* clonal isolates retrieved from the same CF patient, comparing IST439 with (A) IST4113, (B) IST4129 and (C) IST4134. A characteristic bin in the NMR spectrum was used for each metabolite identified. Each square represents the correlation between the metabolite on the head of each column and the metabolite on the head of each row, given by R^2 value (coefficient of determination) resulting from a Pearson correlation analysis in a false color scale (color key at the right). The metabolite correlation map was obtained using MetaboAnalyst 3.0 (Xia et al. 2015).
